# Fibroma of the Ovary; Report of Two Cases

**Published:** 1904-12

**Authors:** F. W. McGuire

**Affiliations:** Buffalo, N. Y.


### Fibroma of the Ovary; Report of Two Cases.

By F. W. McGUIRE, M. D., Buffalo, N. Y.

FIBROMA of the ovary has been considered one of the rarest forms of ovarian new growths. While past literature contains very little on this subject quite recently a number of articles have appeared. Indeed, these cases would seem to be by no means as rare as heretofore has been believed. The history of my case is not as complete as I could wish, the woman being a foreigner of very limited intelligence. However, I was able to glean the following data:

Mrs. M. Z., widow, aged 56 years, was seen by Dr. Schroeter, who was told that three other physicians had pronounced her case one of ascites. Dr. Schroeter, however, examined her abdomen and found that ascites was accompained by a large nodular tumor. She was sent to the German Hospital for operation. Her previous illness consisted only of childbirths of which she had seven, but no miscarriages. Three of her children died during infancy; the remainder are living and healthy. Menstruation began at eighteen, was regular but painful until the first child was born, ceasing at the age of 54, at which time she claims to have "caught cold," this being the beginning of her present illness. Shortly after the cessation of menstruation she noticed her abdomen was increasing in size, which has gradually continued until this record was made.

Upon examination the abdomen was found to contain fluid; also a large irregular tumor, extending above the umbilicus, of pelvic origin. It was somewhat movable and by bimanual examination the uterus seemed to move independently of the tumor. The uterus was three and a half inches in depth.

Operation.—On October 12, 1903, when the peritoneal cavity was opened a large quantity of ascetic fluid escaped and a large irregular tumor presented, which was found to arise from the left side of the pelvis. There were a few adhesions to the small intestines and a pronounced attachment at the bottom of the pelvis. The peritoneum was engorged, the bloodvessels were dilated, right ovary was in a state of atrophy, the appendix seemed normal, although longer than usual, while the uterus appeared about normal in size. I removed the left tube in connection with the tumor, the latter being attached to the growth. I closed the abdomen by layer sutures reinforced by silkworm gut. Her convalescence was uneventful. The gross appearance of the tumor is irregular and nodular to which is attached the Fallopian tube and a portion of the broad ligament. Its weight is eleven pounds, length, 8¼ inches; width, 8 inches, and thickness, 5¾ inches; consistency, firm and elastic.

Pathological Report.—Dr. Herbert U. Williams, pathologist at the University of Buffalo, reports as follows: diagnosis, fibroma, moderately soft. There is no admixture of smooth muscle. The main constituents of the growth are white fibrous tissue and connective cells. The fibers occur in bundles, very irregular in size and direction. The cells are in part spindle-shaped, like connective tissue cells; in part they are smaller, round with round nuclei and appear like young connective tissue cells. No mitosis was seen. In the more richly cellular parts, the cells occur in groups of large size; these parts resemble sarcoma closely, and this tumor is thus related to sarcoma. I should not, however, expect it to produce metastasis or show other malignant tendencies.

The superficial part of the tumor is dense, the remainder being looser; there are many small cavities filled with gelatinous material; these cavities appear to have arisen by degeneration of the substance of the tumor, the jelly like formation being one of the varieties of mucin. Hemorrhage has certainly contributed to the formation of these cysts. Some of them contain blood pigment, fibrin, leukocytes, and even well preserved red blood corpuscles. There has been some hemorrhage into the solid parts of the tumor; it is well supplied with bloodvessels and some parts are very vascular.

The most interesting features in this case are the ascites and weight of the tumor. Ascites occurs in about 40 per cent. of ovarian fibroma, more frequently in the larger than the smaller variety. It is peculiar that fluid should be found in some cases and not in others and why it should be found in any case we are still at a loss to determine. Some maintain that it is due to the bruising of the peritoneum, but it is difficult to see how a small tumor could inflict sufficient injury to the peritoneum to cause the amount of fluid that is seen in some instances. If it were due to excessive mobility we would expect to find it associated with small cystic and dermoid ovarian growths. Furthermore, it has been reported several times at operation that no changes existed in the peritoneum. In this case, however, the peritoneum was engorged and the bloodvessels dilated although there was no apparent thickening.

The fact that some of these are mistaken for ascites should always put us on our guard when the ascites is of obscure origin. Peterson reports a case that was sent to the hospital for ascites which was tapped sixty-five times. I saw my case three months after operation and there was no return of the ascites.

From my examination of the literature, the weight, eleven pounds, appears somewhat unusual. Peterson, in a report of eighty cases, finds one case (Fleishmann's), weighing twenty pounds, one fifteen, one twelve, one eight, five cases each of seven, six, four pounds, seven, three pounds, four two pounds and sixteen one pound or under; in the remaining thirty-eight cases no mention is made of the weight, which were presumably of small size.

Since writing the foregoing my brother, Dr. E. R. McGuire, had a patient with an ovarian fibroma accompanied by ascites. She was an unmarried woman, aged 40 years. There was nothing of interest in this case until a year ago when she consulted her family physician for metrorrhagia and menorrhagia. She demurred so strongly to a physical examination that he was compelled to treat her empirically for almost a year, with no relief of her symptoms. Matters thus progressed until October 4, 1904, when he was hurriedly called to her home and found her suffering with severe pain that required half a grain of morphine to relieve. Physical examination at this time revealed the presence of a large tumor of apparent pelvic origin extending well up to the umbilicus. A blood count at this date showed reds 3,000,000, whites 9,000. The patient was operated on October 14, 1904. When the abdomen was opened quite a quantity of ascetic fluid escaped. A tumor of the left ovary presented, which was removed together with the left tube. The right ovary contained a cyst, about the size of an orange, which necessitated its complete removal. The peritoneum was markedly congested, there were some adhesions between the tumor and intestines, presenting evidence of peritonitis. A later examination of the tumor on left side showed it to be made up of four distinct masses, three of which on section proved to be fibromata, with small hemorrhagic infarcts scattered throughout. The other tumor, located posteriorly, proved to be cystic.

The weight of the tumor after the escape of the cystic fluid was three pounds. This case is of interest on account of the cystic condition accompanying the fibroid.

1175 Main Street.

